# Selection, Isolation, and Characterization of Bacteriophage MA9V-3 from *Chryseobacterium indologenes* MA9

**DOI:** 10.3390/v18040413

**Published:** 2026-03-27

**Authors:** Jinmei Chai, Qian Zhou, Yangjian Xiang, He Zou, Yunlin Wei

**Affiliations:** Faculty of Life Science and Technology, Kunming University of Science and Technology, Kunming 650500, China; 15908743839@163.com (J.C.); 17687026368@163.com (Q.Z.); xiangyangjian163@163.com (Y.X.); 18381307626@163.com (H.Z.)

**Keywords:** Notoginseng, root rot disease, *Chryseobacterium indologenes*, bacteriophage, genomics, phylogenetic analysis

## Abstract

*Chryseobacterium indologenes* MA9 is a causative agent of root rot disease in *Panax notoginseng (P. notoginseng)*, with its high incidence being a major manifestation of continuous cropping barriers, severely hindering the sustainable development of the *P. notoginseng* industry. In this study, a novel lytic bacteriophage, MA9V-3, was isolated from wastewater, targeting *C. indologenes* MA9. The phage produced clear plaques, ranging from 1 to 3 mm in diameter, with a surrounding halo. Phage MA9V-3 achieved an adsorption rate of up to 80% after 30 min of contact with *C. indologenes* MA9, a latent period of approximately 40 min, and an average burst-size if 160 PFU/cell. Transmission electron microscopy revealed that phage MA9V-3 possesses an icosahedral head and a contractile tail, exhibiting a typical myovirus-like morphology. According to the latest ICTV taxonomy, MA9V-3 belongs to the class *Caudoviricetes*, and the phage’s biocontrol efficacy and inhibitory capacity were evaluated at different multiplicity of infection (MOI s). The results showed that the highest titer recorded at 1.6 × 10^10^ PFU/mL. Whole-genome sequencing revealed that MA9V-3 is a double-stranded circular DNA virus, with a genome length of 103,203 bp, GC content of 34.29%, and 150 open reading frames (ORFs), one of which is related to *tRNA*. Only 13 of these ORFs encode known functional sequences, likely due to the limited available gene data for such phages in the database, with additional details on hypothetical proteins yet to be uncovered. Comparative database analysis confirmed that the phage genome contains no antibiotic resistance or toxin-related genes. Phage therapy experiments were performed using MA9V-3 and two other phages screened in our laboratory. The experimental results showed that phage MA9V-3 may be a potential candidate for effectively controlling the infection of *Panax notoginseng* by *C. indologenes* MA9, and offering valuable insights into the potential application of phage therapy for managing bacterial plant diseases.

## 1. Introduction

*Panax notoginseng* (Burk.) F.H. Chen is a valuable traditional Chinese medicinal herb primarily cultivated in Yunnan Province and Guangxi, China [1]. Owing to its preference for warm, shaded, and humid environments, *P. notoginseng* is highly susceptible to various soil-borne diseases, among which root rot is one of the most destructive, severely reducing yield and quality and threatening sustainable production. As the duration of cultivation increases, the frequency, variety, and severity of these diseases escalate, with root rot being one of the most destructive diseases impacting *P. notoginseng*. Root rot significantly diminishes both yield and quality, threatening the sustainability of the *P. notoginseng* industry [2].

The root rot disease complex (RRDC) of *P. notoginseng* involves multiple pathogens [1], including fungi such as *Fusarium* spp., *Rhizoctonia* spp., and *southern blight pathogens* [3], as well as bacteria such as *Pseudomonas* spp. [4,5]. *Chryseobacterium indologenes* has recently been identified as an important bacterial pathogen of root rot in *P. notoginseng*, with pathogenicity confirmed through isolation and bioassays [6]. This bacterium has also been associated with crown and root rot in other crops, highlighting its potential agricultural risk. The persistence of root rot pathogens in soil and the need for crop rotation further complicate disease management and increase production costs [7].

Bacteriophages have gained increasing attention as promising biocontrol agents for plant bacterial diseases owing to their high host specificity, self-replicating nature, and minimal environmental impact compared with chemical bactericides [8,9,10,11,12]. Unlike broad-spectrum antimicrobial compounds, lytic phages selectively infect and lyse target bacteria without disturbing beneficial microbiota, making them particularly attractive for sustainable agriculture and medicinal plant production systems [9,13]. Over the past decade, extensive research has demonstrated the feasibility of phage-based strategies in controlling various phytopathogenic bacteria, including *Xanthomonas*, *Pectobacterium*, *Erwinia*, and *Ralstonia* species [14,15,16,17]. These studies have highlighted not only the direct bacteriolytic activity of phages but also their ability to suppress pathogen populations in planta and reduce disease severity under greenhouse and, in some cases, field conditions.

Recent advances have further emphasized the importance of phage cocktails targeting multiple bacterial receptors to enhance antibacterial efficacy and delay the emergence of phage-resistant mutants [11,12,18]. By imposing higher evolutionary costs on bacterial adaptation, multi-phage formulations may reduce the likelihood of resistance development and improve treatment stability. Moreover, integrated approaches combining phages with other biological control agents or reduced chemical inputs have been proposed within the framework of sustainable and integrated plant protection strategies.

Despite these promising developments, several challenges still limit the large-scale application of phages in plant disease management [18]. Environmental factors such as ultraviolet radiation, temperature fluctuations, pH variability, and limited persistence on plant surfaces can reduce phage viability in field conditions. In addition, regulatory considerations and formulation optimization remain key obstacles to commercialization. Therefore, continuous exploration of novel, environmentally adaptable, and genetically safe lytic phages is essential for advancing phage-based biocontrol strategies [12,19].

In this study, a lytic bacteriophage, MA9V-3, targeting *C. indologenes* MA9 was isolated and characterized. Its biological properties and genomic features were analyzed, and its antibacterial efficacy was evaluated both in vitro and in vivo using single and combined phage treatments. These findings provide a basis for developing phage-based strategies to manage bacterial root rot of *P. notoginseng*.

## 2. Materials and Methods

### 2.1. Bacterial Strains and Growth Conditions

The *C. indologenes* strain MA9 (CP075170), which was isolated from *Panax notoginseng* root rot, was provided by Yunnan Agricultural University. The *C. indologenes* strain MA9 was cultured at 28 °C with shaking at 160 rpm in optimized Nutrient Agar (NA) medium, which contains 10 g of peptone, 5 g of sodium chloride, and 3 g of beef extract. sodium chloride was supplied by Tianjin Damao Chemical Reagent Factory (Tianjin, China). In addition, peptone was obtained from Oxoid Ltd. (Basingstoke, UK), and beef extract was sourced from Angel Yeast Co., Ltd. (Yichang, China).

### 2.2. Isolation, Purification, and Storage of Phages

The phages used in this study were isolated from various locations as follows: (1) Soil collected in April 2024 from a wooded area near Kunming University of Science and Technology, located in Chenggong District, Kunming, Yunnan Province. (2) A mixed sample of river sand and water collected in April 2024 from the Laoyu River, also near Kunming University of Science and Technology in Chenggong District. (3) Sewage samples collected in May 2024 from multiple hospitals in Kunming, Yunnan Province, including: the Chenggong District Branch of Yunnan Provincial Hospital of Traditional Chinese Medicine, the Chenggong District Branch of the First Affiliated Hospital of Kunming Medical University, Chenggong District Women and Children’s Health Center, Yan’an Hospital, the Affiliated Stomatology Hospital of Kunming Medical University, and the Third Affiliated Hospital of Kunming Medical University (Yunnan Provincial Cancer Hospital). We used a virus enrichment method combined with the double agar plate technique [20], to isolate phages from mixed soil eluates and hospital wastewater samples. To begin, the mixed sample for phage isolation was filtered through sterile gauze to remove solid impurities. The filtered water sample was then mixed with SM buffer (100 mM NaCl, 10 mM MgSO_4_, 50 mM Tris-HCl, pH 7.5, and 0.01% gelatin) in a 1:1 ratio and allowed to stand overnight. Afterward, this mixture was combined with an equal volume of *C. indologenes* MA9 bacterial culture at the logarithmic growth phase (OD600 = 0.6–0.8) and incubated overnight at 28 °C on a horizontal shaker (160 rpm). The resulting mixture was transferred to a 50 mL centrifuge tube and centrifuged at 15,000× *g* for 15 min at 4 °C. The supernatant was then filtered through a 0.22 μm membrane filter. Ten milliliters of the filtrate was added to 100 mL of *C. indologenes* MA9 bacterial culture, and the mixture was incubated overnight at 28 °C on the horizontal shaker (160 rpm). This step was repeated 2–3 times to further enrich the phage MA9. The phage filtrate was then serially diluted using a 10-fold dilution method and mixed with 300 μL of *C. indologenes* MA9 culture. After incubating the mixture for 15 min at 28 °C, 4.5 mL of semi-solid NA medium was added, and the solution was poured onto the surface of an NA solid agar plate. Single plaques were picked from the NA double-layer agar plate and added to a logarithmic phase bacterial culture for further phage propagation, which was then incubated overnight at 28 °C on a horizontal shaker (160 rpm). After incubation, the phage solution was centrifuged at 15,000× *g* for 15 min, and the supernatant was filtered through a 0.22 μm membrane. The filtrate was serially diluted and transferred to double-layer agar plates for further isolation. This process was repeated until the plaque size and morphology became consistent, yielding purified phage stocks, which were stored at 4 °C.

### 2.3. Phage Host Range

The host range of phage MA9V-3 was tested using Double-layer agar plaque assay. The procedure was as follows: First, bacterial cultures were prepared in the logarithmic growth phase, including seven strains of *Chryseobacterium indologenes* (*Chryseobacterium indologenes* MA9, a standard strain purchased from the strain preservation center, and six isolated strains numbered 01 to 06 from the laboratory), one strain of *Pseudomonas syringae* (*Pseudomonas syringae pv. syringae*, purchased from the China General Microbiological Culture Collection Center), two strains of *Escherichia coli* (*Escherichia coli* ATCC 11303 and *Escherichia coli* BW25113, both purchased from the preservation center), and one strain of *Serratia marcescens* (*Serratia marcescens* KMR-3, a strain preserved in the laboratory). For each bacterial strain, 300 μL of the bacterial culture was transferred into a 5 mL sterile EP tube, followed by the addition of 4–5 mL of pre-warmed semi-solid agar medium. After mixing, the suspension was evenly spread on the surface of an agar plate. Once the plate had dried, 10 μL of phage solution was added to the center of the plate. After the phage drop dried, the plate was inverted and incubated overnight in a constant temperature incubator. The next day, the presence of inhibition zones on the agar plate was observed to determine whether the phage could lyse a variety of host bacteria.

### 2.4. Determination of the Optimal Multiplicity of Infection (MOI)

The experimental method followed is based on the protocol described in the literature [21]. To determine the optimal MOI, phage stock with a known titer and host cells in the logarithmic growth phase were mixed at MOI gradients ranging from 0.001 to 10. The mixture was incubated for 12 h at 28 °C on a shaker (160 rpm). After incubation, the enriched sample was centrifuged at 15,000× *g* for 15 min at 4 °C. The supernatant was then filtered through a 0.22 μm membrane, and the phage titer was measured using the double-agar plate method. This procedure was repeated three times to ensure accuracy.

### 2.5. Phage Adsorption Assay

The method for determining this was slightly modified from the procedure described in the literature [22]. To assess phage adsorption, 1 mL of phage dilution at the optimal MOI of 1 was used to infect 9 mL of *C. indologenes* MA9 host cells in the logarithmic growth phase. The mixture was then transferred to a 50 mL sterile conical flask and incubated at 28 °C. At 4-min intervals, approximately 100 μL of the culture was withdrawn and mixed with 1.9 mL of pre-chilled NA medium. The mixture was then centrifuged at 15,000× *g* for 15 min at 4 °C to remove the phage-bacteria complexes. The free phage titer in the supernatant was determined using the double-agar plate method. This experiment was repeated three times to ensure reproducibility.

### 2.6. One-Step Growth Curve

The parameters for the one-step growth curve were determined using the protocol described previously [23], Culture the host bacterium *C. indologenes* MA9 to the logarithmic growth phase. Add phage at the optimal MOI = 1 ratio, allow adsorption at room temperature for 15 min, then centrifuge the mixture at 8000× *g*, 4 °C for 5 min. Retain the pellet, resuspend and wash it with pre-cooled NA liquid medium at 4 °C, centrifuge again, and repeat washing three times. Handle gently during washing to avoid disturbing the cells and ensure removal of free phage particles. Add 50 mL of NA liquid medium pre-incubated at 28 °C to the final pellet, and incubate at 28 °C, 160 rpm in a constant-temperature shaker. Collect samples every 10 min. After sampling, centrifuge at 15,000× *g*, 4 °C for 5 min, filter the supernatant through a 0.22 μm sterile filter membrane, and store at 4 °C. After 150 min of sampling, dilute all samples to appropriate gradients using SM buffer by tenfold serial dilution. Determine the phage titer at each time point using the double-layer agar plate method. Burst size is defined as the number of plaque-forming units (PFU) at the maximum phage titer divided by the number of host bacterial cells infected at the initial stage of infection. Plot the phage titer (logarithmic scale) on the y-axis against time on the x-axis. Each experiment is repeated three times.

### 2.7. Thermal and pH Stability

The experimental procedure was carried out as recommended, with slight modifications [22,24]. For thermal stability, 1 mL of phage MA9V-3 lysate (10^10^ PFU/mL) was treated at temperatures of 4 °C, 28 °C, 40 °C, 50 °C, 60 °C, and 70 °C for 1 h, followed by a 10-min incubation at room temperature. After the treatment, the samples were serially diluted by a ten-fold gradient and the phage survival rate was determined using the double-agar plate method. For pH stability, different buffers with pH values ranging from 3 to 13 were prepared. 100 μL of phage solution was added to 900 μL of each pH buffer, mixed, and incubated at 28 °C for 1 h. Phage titers were then assessed using the same procedure as for the thermal stability tests. All experiments were repeated three times to ensure reproducibility.

### 2.8. Phage Transmission Electron Microscopy (TEM)

Transmission electron microscopy (TEM) was used to visualize the morphology and structure of purified phage particles. Phage particles, purified by density gradient centrifugation, were adsorbed onto carbon-coated copper grids for 10 min. After adsorption, the grids were negatively stained with 1% phosphotungstic acid. Multiple samples were examined using a Hitachi HT7820 transmission electron microscope, under an operating voltage of 120 kV [25]. The transmission electron microscope (Hitachi HT7820) was manufactured by Hitachi Ltd. (Tokyo, Japan)

### 2.9. Phage DNA Extraction, Sequencing, and Bioinformatics Analysis

Phage genomic DNA was extracted using the OMEGA DNA extraction kit for large-scale genomic DNA isolation. The phage DNA concentration was measured using a NanoDrop spectrophotometer (BIO-DL, Shanghai Baoyu De Scientific Instrument Co., Ltd. Shanghai, China), and the quality was assessed via 1% agarose gel electrophoresis. Phage genome sequencing was performed by Genewiz Biotech Co., Ltd. (Shanghai, China). The FastQC software version 0.11.9 was used to assess the raw sequencing data, evaluating metrics such as base quality scores, sequence length distribution, and GC content. The data were then processed using Trimmomatic to remove low-quality reads, resulting in high-quality data for downstream analysis. De novo assembly of the sequencing data was carried out using SPAdes https://gitcode.com/gh_mirrors/sp/spades (accessed on 25 January 2026), software, followed by sequence correction using PrInSeS-G.

Primers were designed based on the assembled sequences, and PCR amplification was performed to verify the accuracy of the sequence assembly by assessing the size of the PCR products. Genome annotation was carried out using Prokka, and repetitive sequences were identified using RepeatMasker. Functional annotation of the gene and protein sequences was performed through multiple database comparisons using NCBI Blast+ https://www.ncbi.nlm.nih.gov (accessed on 25 January 2026). Additionally, genes related to antibiotic resistance and virulence factors were screened by querying the Comprehensive Antibiotic Resistance Database (CARD, https://card.mcmaster.ca, accessed on 30 September 2022) and the Virulence Factor Database (VFDB, https://www.mgc.ac.cn/cgi-bin/VFs/v5/main.cgi, accessed on 20 March 2026). The complete phage genome map was visualized using CGView (https://cgview.ca/, accessed on 25 January 2026). Evolutionary analysis was performed by constructing a phylogetic tree using the four open reading frames (ORFs) of phage MA9V-3, with nucleotide sequence alignment conducted via BLAST. Evolutionary analysis was performed by constructing a phylogenetic tree based on the amino acid sequences of four conserved ORFs of phage MA9V-3, with protein sequence alignment performed using BLASTp https://blast.ncbi.nlm.nih.gov/ (accessed on 25 January 2026).

### 2.10. In Vitro Lysis Assay of Phage MA9V-3

To assess the effect of phage MA9V-3 on the growth of *C. indologenes* MA9, a lysis curve was constructed. The preserved bacterial strains were first activated by streaking onto NA solid medium and incubating at 28 °C. The process was repeated three times, and a single colony was transferred to a liquid NA medium and cultured in a test tube at 28 °C with shaking at 160 rpm for 24 h. After incubation, 1% of the bacterial culture from the test tube was inoculated into a 100 mL conical flask and grown at 28 °C with shaking at 160 rpm until the logarithmic growth phase was reached. The culture was then transferred at 1% into a 300 mL conical flask and further grown to the logarithmic growth phase. The culture was aliquoted into six sterile 50 mL conical flasks. Phage MA9V-3 was added to each flask at various MOIs of 0, 0.001, 0.01, 0.1, 1, and 10, with the control group receiving an MOI of 0. All flasks were incubated at 28 °C with shaking at 160 rpm. At hourly intervals, 200 μL of the culture was sampled, and the OD595 was measured using a microplate reader. Each group was tested in triplicate, and the experiment was repeated three times to ensure reproducibility.

### 2.11. In Vivo Biocontrol Assay of Bacteriophage in Panax notoginseng

The virulence assessment was divided into experiments: in vivo phage treatment experiment. In Vivo Phage Treatment Experiment: Healthy, two-year-old *Panax notoginseng* plants were transplanted into pots and allowed to stabilize before the experiment began. To minimize the influence of complex soil microbial communities, the potting soil was sterilized by high-pressure steam sterilization before transplantation. Two-year-old healthy *Panax notoginseng* plants were transplanted into sterilized soil and allowed to acclimate for 7 days under suitable conditions, during which soil moisture was maintained and direct sunlight was avoided. After the plants stabilized, inoculation and phage treatment experiments were conducted.

The experimental grouping and treatment procedures are summarized. Each treatment was applied at a volume of 50 mL per pot. The experimental groups included: (i) negative control (NC), treated with sterile NA liquid medium; (ii) *Chryseobacterium indologenes* MA9 inoculation group (50 mL/pot); (iii) MA9 + phage MA9V-3 treatment group (50 mL/pot); and (iv) MA9 + phage cocktail (MA9V-1/MA9V-2/MA9V-3) treatment group (50 mL/pot). For root inoculation, *C. indologenes* MA9 cultures at the logarithmic growth phase were used. The phage-treated groups received either a single phage or a phage cocktail together with the bacterial suspension. To avoid excessive water accumulation and possible root suffocation, bacterial suspensions and phage lysates were applied once every three days rather than daily. Plant growth status and disease symptoms were monitored daily. Each experiment was performed in triplicate to ensure reproducibility.

Disease incidence (%) = (number of symptomatic plants/total number of plants) × 100%.

## 3. Results

### 3.1. Morphology of Phage MA9V-3

**Figure 1:**
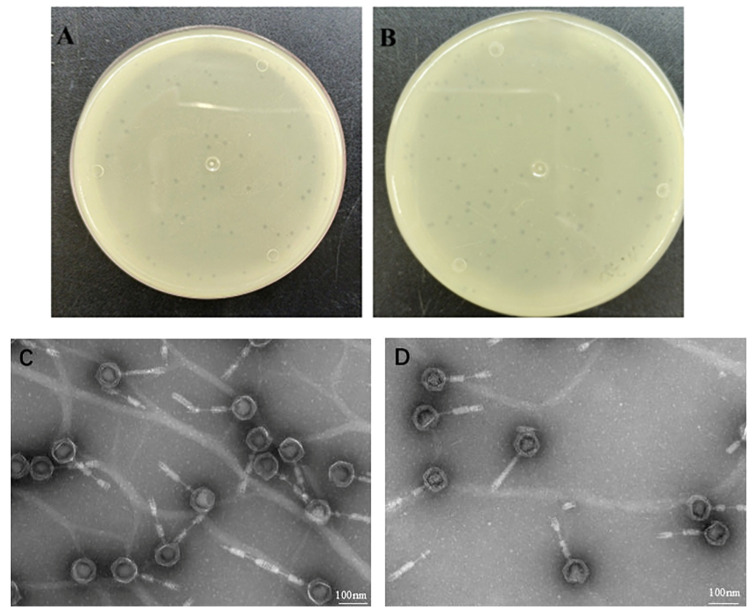
Morphological characteristics of *Chryseobacterium* indologenes phage MA9V-3. (**A**,**B**) Phage plaque morphology of phage MA9V-3 on an NA double layer agar plate and (**C**,**D**) TEM image of phage MA9V-3.

Using *C. indologenes* MA9 as the host, a phage was isolated from a mixed hospital wastewater using the double-agar plate method. This phage was named MA9V-3. The results showed that phage MA9V-3 formed uniform plaques on the double-agar plate, with a diameter of approximately 1–3 mm. The centers of the plaques were clear and transparent, surrounded by a distinct halo (Figure 1A,B). Transmission electron microscopy (Figure 1C,D) revealed that MA9V-3 has an icosahedral “head” with a 20-sided structure, and a contractile tail. The phage had an average total length of 248.5 ± 0.30 nm, an average head diameter of 84.9 ± 0.30 nm, and an average tail length of 163.6 ± 0.30 nm (mean ± SD, *n* = 20).

### 3.2. Biological Characteristics of Phage MA9V-3

**Figure 2:**
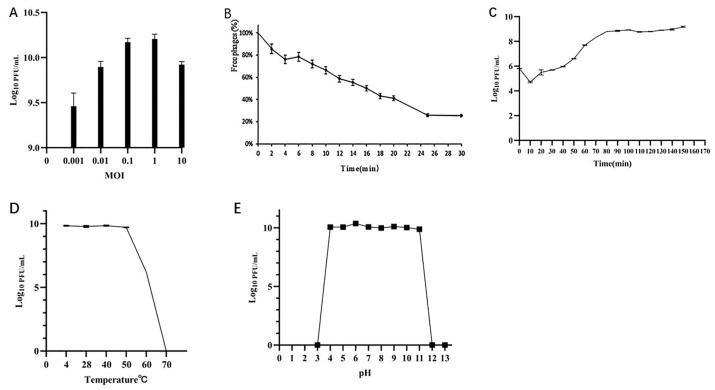
Biological features of phage MA9V-3. (**A**) Titers at different MOIs; (**B**) Adsorption curve; (**C**) One-step growth curve; (**D**) Thermal stability analysis; (**E**) pH stability analysis.

As shown in (Figure 2A), the titer of phage MA9V-3 was 1.6 × 10^10^ PFU/mL, with the highest titer observed at an optimal MOI of 1. This indicates that the highest number of progeny phages, approximately 10^10^ PFU/mL, was produced when the phage and host bacteria were mixed at this ratio. The phage adsorption curve reflects the ability of the phage to bind with the host. As shown in (Figure 2B), after 20 min, only 40% of the free phages remained, while approximately 60% of the phages had adsorbed onto the host. The one-step growth curve of phage MA9V-3, as shown in (Figure 2C), reflects its lytic capacity. The latent period of phage MA9V-3 is approximately 40 min, during which the phage population grows slowly. The lytic phase lasts for 50 min, followed by a stationary phase after 90 min, where the number of released progeny phages peaks at 160 PFU/cell. The effect of temperature and pH on phage activity was evaluated by counting plaques on double-agar plates and determining the phage titer under various conditions. As shown in Figure 2D, phage MA9V-3 remained active across a temperature range of 20 °C to 50 °C. Although the titer at 40 °C appeared numerically higher than at other tested temperatures, no statistically significant differences were observed among these conditions. The optimal growth temperature of the host bacterium was 28 °C. The phage was completely inactivated at temperatures above 60 °C.Regarding pH stability (Figure 2E), phage MA9V-3 showed no detectable activity at pH ≤ 3 or pH ≥ 12. The phage retained activity within the pH range of 4.0 to 11.0. Although the titer at pH 6.0 was numerically greater than at other tested pH values, no statistically significant differences were detected among the active pH conditions. Therefore, pH 6.0 is described as the condition under which relatively higher activity was observed within the tested range.

The results show that MA9V-3 infects *Chryseobacterium indologenes* MA9, 02, and 06, but does not infect *C. indologenes* ATCC 29897, 01, 03, 04, or 05, nor *Pseudomonas syringae* pv. *syringae*, *Escherichia coli* ATCC11303, or *E. coli* BW25113. These results demonstrate that MA9V-3 exhibits strain-specific host specificity within *C. indologenes*. n the table, “+” denotes the occurrence of lysis, while “−” indicates the absence of lysis (Table 1).

### 3.3. Genome Sequencing Analysis of Phage MA9V-3

The genome sequence information of the bacteriophage has been submitted to the GenBank database, with the accession number: PV245944. The complete genome sequence of phage MA9V-3 was obtained through genome sequencing, with a total length of 103,203 bp. The GC content is 34.29%, and the AT content is 65.71%. Alignment results showed that phage MA9V-3 shares the highest similarity with the lactic acid bacteria phages *Lactobacillus* phage SAC12B (GenBank accession no. NC_048754.1) and *Lactobacillus* phage 521B (GenBank accession no. NC_048752.1), although the coverage was only 4%. The phage MA9V-3 genome contains a total of 150 open reading frames (ORFs), with the longest ORF being 4380 base pairs and the shortest only 98 base pairs. One of these ORFs is associated with tRNA. Of the 150 ORFs, 137 are hypothetical proteins with unknown functions, while 13 are known functional coding sequences (CDS). As shown in Table 2, the first four ORFs are associated with DNA replication, while ORFs 81, 84, 93, and 96 are related to DNA repair. The genes related to protein synthesis include ORFs 55, 62, and 83, with ORF 55 encoding *tmRNA* (transfer-messenger RNA, *SsrA*). ORF 94 encodes GTP cyclohydrolase I, a rate-limiting enzyme in the tetrahydrobiopterin (BH4) biosynthesis pathway. This enzyme is crucial for several biological processes, including the synthesis of neurotransmitters and regulation of oxidative stress in mammals. Its role in phages remains unclear. ORF 98 encodes 7-carboxy-7-deazaguanine synthase (*queE*), which plays a key role in the folate synthesis pathway in bacteria and archaea.

Although many structural proteins were originally annotated as hypothetical, conserved domain analysis identified several ORFs with predicted structural roles, including: Major capsid protein-like domains; Portal protein-associated motifs; Tail fiber-related β-helix folds; Baseplate assembly-like domains. These genes are organized in clusters, consistent with the classical head–tail morphogenesis module observed in Caudoviricetes phages. The structural gene cluster is positioned downstream of replication genes, suggesting temporal regulation of early (replication) and late (assembly) transcription. Several hypothetical proteins located within the structural gene cluster were predicted to contain β-helix structural motifs commonly associated with tailspike depolymerases. Given the observed halo surrounding plaques, these proteins may function as capsular polysaccharide-degrading enzymes. Although sequence identity to known depolymerases was relatively low (<30%), structural homology modeling indicated similarity to carbohydrate-binding or polysaccharide lyase folds. These findings support the hypothesis that MA9V-3 encodes capsule-degrading activity, potentially enhancing infection efficiency against encapsulated *C. indologenes*. Of the 137 initially annotated hypothetical proteins, conserved domain searches revealed that: Several contain helix-turn-helix (HTH) DNA-binding motifs; Some exhibit predicted transmembrane helices suggestive of holin-like proteins; Others possess enzymatic domains associated with nucleotide metabolism or protein–protein interaction. This refined annotation significantly reduces the proportion of functionally uncharacterized proteins and supports the modular genome architecture of MA9V-3. To evaluate the suitability of MA9V-3 for biocontrol applications, the genome was screened against the CARD and VFDB databases using thresholds of E-value < 1 × 10^−5^ and sequence identity >30%. No antibiotic resistance genes, virulence factors, integrases, or lysogeny-associated repressors were detected. The absence of integrase genes and repressor proteins further supports the classification of MA9V-3 as a strictly lytic phage. These findings suggest that MA9V-3 does not pose a risk of horizontal transfer of antibiotic resistance or pathogenicity determinants, supporting its potential use in biological control.

**Figure 3:**
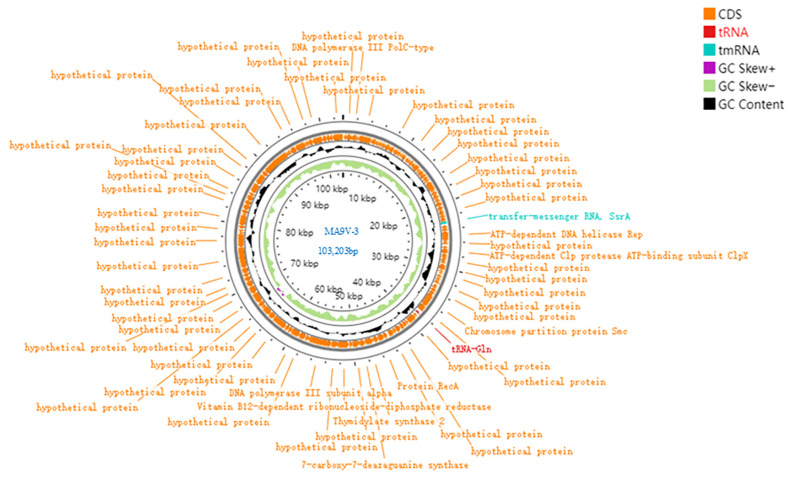
Whole genome map of MA9V-3.

The entire genome map of phage MA9V-3 was generated using the online tool ProKsee (v1.14.6)(Figure 3). The orange color represents all coding sequence ORFs, while the purple and light green regions indicate GC skew. Black indicates GC content, red represents *tRNA*, and blue represents *tmRNA*.

### 3.4. Phylogenetic Tree of Phage MA9V-3

**Figure 4:**
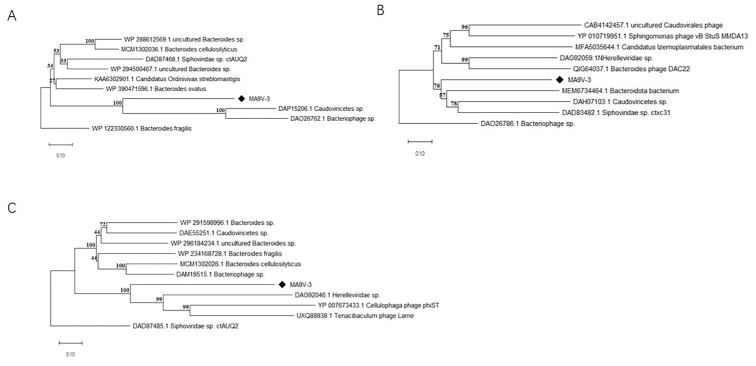
Protein phylogenetic tree of the selected *C. indologenes* phage MA9V-3 and other phages based on deduced amino acid sequences of (**A**) DNA polymerase III PolC-type; (**B**) ATP-dependent DNA helicase Rep; and (**C**) Chromosome partition protein Smccc. The position of phage MA9V-1 in the picture is marked with a black diamond.

Based on the proteome-based phylogenetic framework, we further constructed trees using three representative conserved proteins to rigorously clarify the evolutionary position of phage MA9V-3. Phylogenetic relationships between MA9V-3 and ten reference sequences were inferred from DNA polymerase III PolC-type (ORF004), ATP-dependent DNA helicase Rep (ORF058), and chromosome partition protein Smccc (ORF81) (Figure 4). Across all three phylogenetic reconstructions (Figure 4A–C), MA9V-3 consistently formed a distinct and well-supported lineage, clearly separated from established bacterial and phage clades. In the PolC tree (Figure 4A), MA9V-3 clustered only with two unclassified viral sequences (*Caudoviricetes* sp. and *Bacteriophage* sp.) with maximal bootstrap support (100), forming an independent viral branch that was clearly distant from the large Bacteroides/Bacteroidota bacterial clade. The pronounced phylogenetic separation from host-derived PolC sequences strongly suggests that the MA9V-3 polymerase represents a phage-specific lineage that has evolved independently within viral populations rather than being recently acquired from bacterial hosts. Similarly, in the Rep helicase tree (Figure 4B), although MA9V-3 showed moderate proximity to several phage- and Bacteroidota-associated sequences, it did not embed within any established genus-level cluster. Instead, it occupied a separate branch with moderate-to-strong support, further reinforcing its evolutionary divergence. This topology is consistent with the modular and mosaic evolution typical of phage replication systems, yet the persistent phylogenetic separation of MA9V-3 indicates a distinct evolutionary trajectory. In the Smccc phylogeny (Figure 4C), MA9V-3 grouped within a phage-associated partition protein clade but again maintained a clearly delineated and well-supported sub-branch (bootstrap 99–100), distinct from known Herelleviridae, Cellulophaga phage phiST, and Tenacibaculum phage Larrie lineages. The consistent formation of an independent branch across replication and genome-partition modules highlights the genomic coherence of MA9V-3 while underscoring its phylogenetic uniqueness.

Taken together, these independent gene trees uniformly demonstrate that MA9V-3 does not cluster tightly within any currently defined phage genus or species group. Instead, it persistently forms a separate and strongly supported lineage, indicating that MA9V-3 represents a genetically distinct Bacteroides-associated tailed phage with a unique evolutionary background. The congruent topological pattern across multiple conserved proteins provides robust evidence for its evolutionary novelty and supports the possibility that MA9V-3 may represent a previously uncharacterized lineage within Caudoviricetes. 

### 3.5. Virulence Assessment of Panax notoginseng Roots

**Figure 5:**
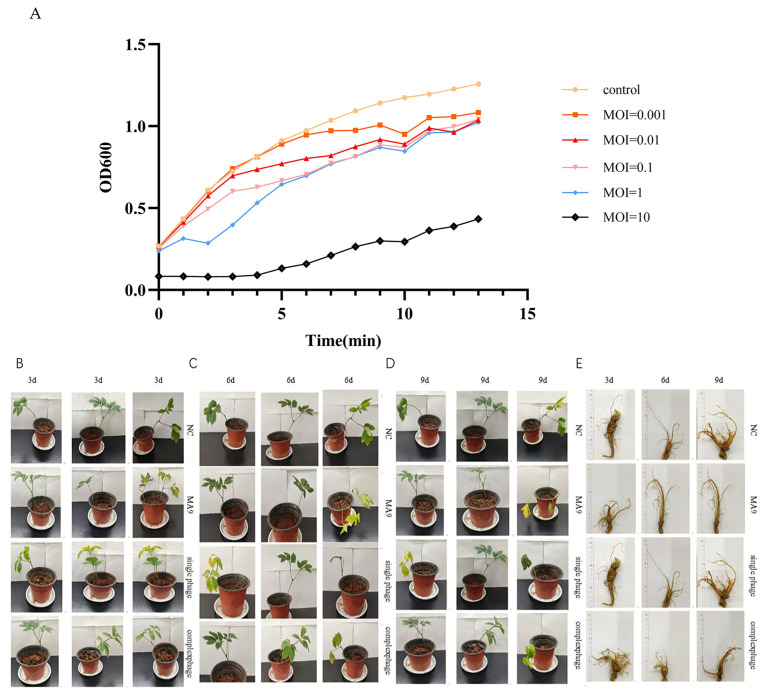
Virulence efficiency of phage MA9V-3 on *C. indologenes* strain MA9. (controlgroup, yellow), 0.001 (orange), 0.01 (red), 0.1 (pink), 1 (blue), 10 (black). (**A**) Bacteriostatic curve of phage MA9V-3 invitro. (**B**) Control effect of experimental treatment on bacteriaafter 3 days; (**C**) Control effect of experimental treatment on bacteria after 6 days; (**D**) Control effect of experimental treatment onbacteria after 9 days; (**E**) The state of panax notoginseng roots after experimental treatment.

To investigate the in vitro lytic activity of phage MA9V-3 against the host *C. indologenes* MA9, we first measured the absorbance at OD600 of *C. indologenes* MA9, both with and without phage MA9V-3, over a 13-h period (Figure 5A). The growth inhibition curve indicates that phage MA9V-3 can inhibit the growth of *C. indologenes* MA9. When cultured alone, the absorbance increased rapidly, reflecting fast bacterial growth. At an MOI of 10, the OD600 value was lower, and the absorbance began to decline between 9–10 h. When the optimal MOI of 1 was used, a decrease in absorbance was observed between 1–2 h, suggesting that phage MA9V-3 exerted the strongest inhibitory effect on the host bacterium at this MOI. In the in vivo phage treatment experiment, on day 3 (Figure 5B), all *Panax notoginseng* plants in the phage-treated groups remained in good condition. By day 6 (Figure 5C), approximately 30% of the plants in the ma9-treated group showed wilting, while about 60% of the plants in the single-phage treatment group exhibited wilting symptoms. By day 9 (Figure 5D), the wilting in both groups became more severe, with 60% of the plants in the ma9-treated group showing wilting, whereas the proportion in the single-phage treatment group exceeded 80%, with plants severely wilted. In contrast, in the mixed-phage treatment group, only about 33% of the plants exhibited mild wilting symptoms by day 9 of cultivation. The group treated with the phage cocktail exhibited significantly improved plant health, characterized by reduced wilting symptoms and less severe root rot, compared to the single-phage treatment group. At the end of the experiment, (Figure 5E) the surface of the roots in the MA9 treatment group was noticeably blackened and exuded bacterial pus during washing, while the roots in the other treatment groups were in a condition similar to the negative control.

## 4. Discussion

In this study, we isolated and characterized a lytic bacteriophage, MA9V-3, infecting *Chryseobacterium indologenes* MA9, a confirmed bacterial pathogen associated with root rot of *Panax notoginseng* [26]. Unlike previous descriptions that primarily emphasized the ecological distribution or debated pathogenic role of *C. indologenes*, our results focus on the phage–host interaction characteristics and their implications for biological control [10].

MA9V-3 formed clear plaques with diameters of 1–3 mm, indicating a strictly lytic lifecycle and strong bacteriolytic capacity. Transmission electron microscopy revealed an icosahedral head and a contractile tail, consistent with tailed phages classified within the class *Caudoviricetes* [27]. The well-defined plaque morphology and absence of turbid zones further support its virulent nature, which is desirable for biocontrol applications [9]. A notable observation was the halo surrounding plaques. This phenomenon is commonly associated with phage-encoded depolymerases that degrade capsular polysaccharides [28]. Given that *C. indologenes* MA9 possesses a prominent capsule, the halo strongly suggests that MA9V-3 likely encodes capsule-degrading enzymes. Such enzymatic activity is particularly advantageous in agricultural settings because capsular polysaccharides often serve as protective barriers against environmental stress and antimicrobial agents [29]. The ability to degrade the capsule may enhance phage penetration, increase infection efficiency, and potentially weaken bacterial virulence in soil environments.

Adsorption assays showed that approximately 60% of phages adsorbed to host cells within 20 min, leaving only 40% free particles. Although the burst size was smaller compared with some reported phages (e.g., Serratia phage LC53), the relatively rapid adsorption rate suggests efficient host recognition. In soil ecosystems, where bacterial density can fluctuate, rapid adsorption is often more critical than large burst size because it enables immediate infection once contact occurs. Interestingly, phage titers varied under different culture conditions despite unchanged capsule formation. This indicates that host physiological status significantly influences infection efficiency. Changes in membrane receptor expression, metabolic activity, or surface polysaccharide conformation under different nutrient conditions may alter phage binding efficiency. These findings highlight the importance of evaluating phage performance under conditions that mimic rhizosphere environments before field application. MA9V-3 remained stable between 20–50 °C and exhibited optimal activity at neutral pH. Considering that *P. notoginseng* is cultivated in subtropical regions with moderate soil temperatures and slightly acidic to neutral soils, these stability characteristics suggest practical compatibility with field conditions. However, inactivation under extreme pH indicates that formulation strategies (e.g., protective carriers or encapsulation) may be required to improve environmental persistence.

Notably, comparative genomic analysis revealed that MA9V-3 shares only 4% genome coverage with its closest relatives currently available in GenBank. Such extremely low sequence similarity indicates that MA9V-3 represents a highly divergent phage and potentially a novel genomic lineage. The absence of closely related reference genomes highlights the limited characterization of *C. indologenes*-infecting phages and underscores the genomic uniqueness of MA9V-3. The high proportion of hypothetical proteins (91.3%) further supports its genomic novelty and suggests the presence of uncharacterized functional modules. Phages with low genomic similarity to known taxa often harbor distinct evolutionary trajectories and may contribute to expanding current phage taxonomy frameworks. Finally, based on comprehensive database comparison analyses, no antibiotic re-sistance genes or known virulence-associated genes were identified in the genome of phage MA9V-3. These findings suggest that MA9V-3 possesses a relatively high level of biosafety for potential biocontrol applications, as it is unlikely to contribute to the dissemination of antibiotic resistance determinants or virulence factors among patho-genic bacteria.

Whole-genome analysis revealed that MA9V-3 possesses a relatively large number of ORFs compared to related phages, suggesting genomic complexity and potential functional diversity. Phylogenetic analysis placed MA9V-3 in a distinct clade together with Caudoviricetes-related sequences, with strong bootstrap support. Notably, the replication-associated genes (e.g, Rep helicase) displayed a mosaic evolutionary pattern, clustering with sequences from diverse bacterial hosts [30]. This mosaicism is characteristic of phage genomes and reflects frequent horizontal gene exchange during evolution [31]. The tight clustering of the Smc/partition protein with well-defined phage SMC modules (bootstrap 99–100) indicates that MA9V-3 contains a typical phage-type partitioning system. Such modules may enhance stable genome maintenance during replication and contribute to efficient propagation within host populations. The presence of these conserved structural and replication elements supports the genetic stability of MA9V-3, an important consideration for biocontrol development [32].

Genomic screening did not detect antibiotic resistance genes or known virulence factors. This is a critical prerequisite for agricultural application, as phage-mediated horizontal gene transfer could otherwise increase pathogen risk [33]. Although unknown genes cannot be entirely excluded, current database comparisons suggest that MA9V-3 is unlikely to contribute to the dissemination of resistance or virulence traits. The combination of strong lytic activity, capsule-degrading potential, environmental adaptability, and absence of undesirable genes supports the suitability of MA9V-3 as a candidate biocontrol agent. However, in vivo experiments were limited to short-term greenhouse assays and small sample sizes. Soil microbial communities are complex, and phage persistence, diffusion, and interaction with native microbiota remain to be evaluated under field conditions. Future studies should focus on: Field-scale trials to validate disease suppression efficacy; Formulation optimization to enhance environmental stability; Phage cocktail design targeting multiple receptors to delay resistance evolution; Investigation of phage–antibiotic or phage–biocontrol microbe synergy [34].

Root rot of *P. notoginseng* is a disease complex involving multiple pathogens. Although MA9V-3 specifically targets *C. indologenes*, integrating phage therapy into a broader integrated disease management framework may reduce bacterial load, weaken synergistic pathogen interactions, and improve overall plant health. Given the increasing restrictions on chemical bactericides and the ecological sensitivity of medicinal plant cultivation systems, phage-based strategies represent a promising sustainable alternative.

## Figures and Tables

**Table 1:** Host range of phage MA9V-3.

Strains	Lysis
*Chryseobacterium indologenes* MA9	+
*Chryseobacterium indologenes* ATCC 29897	(−)
*Chryseobacterium indologenes* 01	(−)
*Chryseobacterium indologenes* 02	+
*Chryseobacterium indologenes* 03	(−)
*Chryseobacterium indologenes* 04	(−)
*Chryseobacterium indologenes* 05	(−)
*Chryseobacterium indologenes* 06	+
*Pseudomonas. Syringae pv. syringae*	(−)
*Escherichia coli* ATCC11303	(−)
*Escherichia coli* BW25113	(−)
*Serratia marcescens* KMR-3	(−)

**Table 2:** Annotated ORF’s in the genome of phage MA9V-3.

ORF	Position	Length/bp	Strand	Description
4	1394..2107	714	-	DNA polymerase III PolC-type
58	24215..25672	1458	-	ATP-dependent DNA helicase Rep
102	50654..52351	1698	-	Vitamin B12-dependent ribonucleoside-diphosphate reductase
105	53483..57040	3558	-	DNA polymerase III subunit alpha
81	34533..36623	2091	-	Chromosome partition protein Smc
84	38559..38873	315	-	DNA-binding protein HU 1
93	44869..45861	993	-	Protein RecA
96	46753..47877	1125	-	Thymidylate synthase 2
55	22766..23124	359	-	transfer-messenger RNA, *SsrA*
62	26938..27777	840	-	ATP-dependent Clp protease ATP-binding subunit ClpX
83	38122..38219	98	-	*tRNA*-Gln
94	45868..46500	633	-	GTP cyclohydrolase 1
98	48087..48854	768	-	7-carboxy-7-deazaguanine synthase

## Data Availability

The data presented in this study are openly available in Mendeley Data at https://data.mendeley.com/datasets/mvfsd52gjw/1 (accessed on 25 January 2026).
